# Supplementary material for: Deep RNA sequencing of intensive care unit patients with COVID-19
Source: Sci Rep. 2022 Sep 21;12:15755. doi: 10.1038/s41598-022-20139-1 (PMC9491252; doi:10.1038/s41598-022-20139-1)
Supplement: Supplementary file 1 — Supplementary Figure 1. [file 41598_2022_20139_MOESM1_ESM.docx]

Supplemental Figure S1: Volcano plot showing genes that have significant gene expression differences between patients who died from COVID-19 and those who survived. Red dots with labels have p<0.05 and greater than 1.5 log_2_ fold change between the groups.
